# Visualization of *Miscanthus* × *giganteus* cell wall deconstruction subjected to dilute acid pretreatment for enhanced enzymatic digestibility

**DOI:** 10.1186/s13068-015-0282-3

**Published:** 2015-07-25

**Authors:** Zhe Ji, Xun Zhang, Zhe Ling, Xia Zhou, Shri Ramaswamy, Feng Xu

**Affiliations:** Beijing Key Laboratory of Lignocellulosic Chemistry, Beijing Forestry University, Beijing, 100083 China; Ministry of Education Key Laboratory of Wooden Material Science and Application, Beijing Forestry University, Tsinghua East Road, Beijing, 100083 China; Department of Bioproducts and Biosystems Engineering, Kaufert Laboratory, University of Minnesota, Saint Paul, MN 55108 USA

**Keywords:** *Miscanthus* × *giganteus*, Dilute acid pretreatment, Enzymatic hydrolysis, Cell wall anatomy, Components distribution

## Abstract

**Background:**

The natural recalcitrance of lignocellulosic plant cell walls resulting from complex arrangement and distribution of heterogeneous components impedes deconstruction of such cell walls. Dilute acid pretreatment (DAP) is an attractive method to overcome the recalcitrant barriers for rendering enzymatic conversion of polysaccharides. In this study, the internodes of *Miscanthus* × *giganteus*, a model bioenergy crop, were subjected to DAP to yield a range of samples with altered cell wall structure and chemistry. The consequent morphological and compositional changes and their possible impact on saccharification efficiency were comprehensively investigated. The use of a series of microscopic and microspectroscopic techniques including fluorescence microscopy (FM), transmission electron microscopy (TEM) and confocal Raman microscopy (CRM)) enabled correlative cell wall structural and chemical information to be obtained.

**Results:**

DAP of *M.* × *giganteus* resulted in solubilization of arabinoxylan and cross-linking hydroxycinnamic acids in a temperature-dependent manner. The optimized pretreatment (1% H_2_SO_4_, 170°C for 30 min) resulted in significant enhancement in the saccharification efficiency (51.20%) of treated samples in 72 h, which amounted to 4.4-fold increase in sugar yield over untreated samples (11.80%). The remarkable improvement could be correlated to a sequence of changes occurring in plant cell walls due to their pretreatment-induced deconstruction, namely, loss in the matrix between neighboring cell walls, selective removal of hemicelluloses, redistribution of phenolic polymers and increased exposure of cellulose. The consequently occurred changes in inner cell wall structure including damaging, increase of porosity and loss of mechanical resistance were also found to enhance enzyme access to cellulose and further sugar yield.

**Conclusions:**

DAP is a highly effective process for improving bioconversion of cellulose to glucose by breaking down the rigidity and resistance of cell walls. The combination of the most relevant microscopic and microanalytical techniques employed in this work provided information crucial for evaluating the influence of anatomical and compositional changes on enhanced enzymatic digestibility.

**Electronic supplementary material:**

The online version of this article (doi:10.1186/s13068-015-0282-3) contains supplementary material, which is available to authorized users.

## Background

The ever-increasing global demand for energy and concerns on our environment are driving research to explore fuel productions from renewable feedstocks to reduce our dependence on scarce petroleum sources [1–3]. The annual solar energy stored in lignocellulosic plant cell walls is nearly ten times that of the total energy used by humans [4]. Thus, the lignocellulosics such as woody materials, agricultural and forestry residues and energy crops have a promising role in large-scale production of liquid transportation fuels and other value-added products. A perennial grass species, *Miscanthus* × *giganteus*, is a promising candidate for generating abundant lignocellulosic biomass at low fertilization requirements compared to plantation species. Studies investigating the effect of genetic and environmental factors on cell wall composition have shown considerable variation among *M.* × *giganteus* genotypes, allowing potential breeding for high-yielding varieties, which can also be easily established and harvested [5].

The process of second-generation bioethanol is based on conversion (hydrolysis) of cellulose into fermentable glucose using polysaccharide-active enzymes. However, the bioconversion is normally hindered by the complex structure and heterogeneous components (cellulose, hemicelluloses, lignin and pectin) distribution in cell walls. Plants have evolved a hierarchical structure during cell wall assembly, which is differentiated into several distinct layers: the shared cell corner middle lamella (Ccml) and compound middle lamella (Cml) that function as a glue between adjoining cells, the thinner primary wall (P) and thicker secondary wall (Sw). In plant cell walls, these layers become lignified. The deposition of the main components within this model is spatially and temporally regulated, and the chemical characteristics differ between cell walls, plant tissues and plant species. Cellulose microfibrils that act as cell wall scaffold are embedded in a matrix of amorphous hemicelluloses and phenolics substances, resulting in a rigid and compact mesh-like structure [6, 7]. Hemicelluloses–lignin matrix that is covalently interwoven with nano-scale structural heterogeneities is a main recalcitrant factor, limiting the penetration and action of enzymes by coating cellulosic microfibrils and nonproductive binding to enzymes [8, 9]. Additionally, the unique hydroxycinnamic acids (HCA) present in grass cell walls play a significant role in cross-linking the chemical polymers into a cohesive network, which further restricts the enzymatic accessibility of cellulose [10–12]. The ultrastructural and compositional complexity of lignocellulosic cell walls is a deterrent to fungal and bacterial attacks.

Due to the natural recalcitrance of lignocellulosic cell walls, a pretreatment is generally required prior to enzymatic hydrolysis to disrupt the compact structure of such cell walls, facilitating access of hydrolyzing enzymes to cellulose. In this regard, pretreatment technologies suited to different lignocellulosic matrices have emerged, which include physical processes and thermochemical and biological treatments [13–16]. Thermochemical pretreatment using acids under low severity are considered as a promising technology for reducing the recalcitrance of the lignocellulosic biomass. In this process, it depolymerizes and solubilizes vast hemicelluloses and less lignin, leaving behind a cellulose-rich substrate that is more amenable to enzymatic degradation [17]. The oligosaccharides and monosaccharides derived from hemicelluloses can be further utilized for producing high-value chemicals. In the last few decades, progress in elucidating chemical modifications within cell walls following dilute acid pretreatment (DAP) has helped exploit enzymatic hydrolysis for the production of fermentable sugars [16, 18].

However, the mechanism of DAP and the influence of ultrastructural and topochemical factors on biomass saccharification efficiency remain largely unclear, making it difficult to intelligently design and optimize cost-effective pretreatment protocols. A detailed understanding of how plant cell walls respond to a pretreatment will be helpful in the optimization of cell wall deconstruction. The corresponding knowledge of cell wall disruption, creation of pits or holes, distribution of xylans and the fate of phenolics and cellulose that affect the efficiency of enzymatic hydrolysis need to be revealed. In our work, *M.* × *giganteus* samples were subjected to DAP with increasing severity. To understand the relationship in the changes occurring in *M.* × *giganteus* tissues during treatment for enzymatic digestibility, combined chemical, morphological and topochemical studies were undertaken using the most pertinent tools and techniques such as fluorescence microscopy (FM), transmission electron microscopy (TEM) and confocal Raman microscopy (CRM). The obtained comprehensive information will be helpful in developing cost-effective pretreatment strategies and designing effective bioengineering processes for improving sugar yield.

## Results and discussion

### Chemical composition

The chemical composition of *M*. × *giganteus* raw materials and samples subjected to DAP is presented in Table 1. The carbohydrate constituents of native samples accounted for 65.78% of the dry matter (DM), which mainly consisted of glucose (40.85% DM, 62.10% total carbohydrates), xylose (21.28% DM, 32.35% total carbohydrates) and arabinose (2.59% DM, 3.94% total carbohydrates), reflecting the two major polysaccharides present in *M*. × *giganteus*, namely, cellulose and arabinoxylan. In addition, samples contained about 1.0% galactose and mannose (Table 1).

**Table 1:** Effect of dilute acid pretreatment on chemical compositions of *M.* × *giganteus* internode fragments

Samples	Sugar monomers (%)					Total sugar monomers (%)	Phenolics (%)		
	Arabinose	Galactose	Glucose	Xylose	Mannose		Klason lignin	PCA	FA
Untreated	2.59	0.48	40.85	21.28	0.58	65.78	19.27	3.72	2.89
150°C, 1%, 15 min	1.16	0.39	42.45	17.28	0.46	61.74	21.40	3.56	2.64
150°C, 1%, 30 min	0.93	0.36	45.03	13.71	0.24	60.27	22.53	3.35	2.42
160°C, 0.5%, 15 min	0.79	0.43	46.39	13.29	0.57	61.47	21.71	3.05	2.19
160°C, 1%, 15 min	0.58	ND	49.34	9.28	0.46	59.66	23.40	2.96	1.99
170°C, 0.5%, 15 min	0.41	0.51	50.38	9.16	ND	60.46	23.92	2.53	2.31
170°C, 1%, 30 min	0.25	ND	53.39	5.63	0.15	59.42	25.77	1.89	2.03

*ND* not detected.

The pretreatment of *M.* × *giganteus* with dilute sulfuric acid initially resulted in a reduction in substrate mass, which was proportional to the pretreatment severity. Statistical analysis revealed that losses in solid ranged from 11.20% under mild condition (1% H_2_SO_4_, 15 min, 150°C) to 27.97% when pretreated in 1% H_2_SO_4_ for 30 min at 170°C (see Additional file 1: Figure S1). Although each pretreatment variable contributed to solids’ loss, temperature was verified to be the main controlling factor, followed by acidity and then residence time. Comparable treatment parameter trends have been reported for alkali pretreatment of wheat straws [19]. Yet, other studies have reported differences between plant species with regard to their susceptibility to diverse pretreatments [20–22].

The relative content of glucose in the dilute acid treated *M.* × *giganteus* samples gradually increased with pretreatment severity, reaching a maximum value of 53.39% DM (89.85% total carbohydrates) at 170°C, 1% H_2_SO_4_ for 30 min. The relative increase in glucose was ascribed to the dissolution of hemicellulosic polysaccharides, especially xylose and arabinose which, respectively, diminished from 21.28 and 2.59% DM to 5.63 and 0.25% DM, indicating solubilization of arabinoxylan. Mannose and galactose were absent or recovered at concentrations lower than 0.20% after pretreatment. Solubilization of hemicelluloses into oligoxylans of mixed molecular weights following thermochemical pretreatment has been reported elsewhere [23, 24]. Upon DAP, the hydronium ions (H_3_O^+^) released by acids lead to selective hydrolysis of glycosidic linkages in hemicelluloses, releasing acetyl groups and other acid moieties to form acetic and uronic acids. The acids further contribute to additional H_3_O^+^, in turn catalyzing the degradation of hemicellulosic polysaccharides [25, 26]. The ratio of arabinose to xylose diminished by threefold (from 0.12 to 0.04) from an increase in pretreatment severity, suggesting linearization and/or easier solubilization of the xylans with a higher level of substitution.

The Klason lignin content in raw materials was 19.27% DM. It gradually increased in the treated samples along pretreatment severity, resulting from the relative reduction in insoluble carbohydrates. *M*. × *giganteus* contains little quantities of HCA that cross-links arabinoxylans and lignin via ester and ether bonds [27–29]. DAP decreased *p*-coumaric acid (PCA) and ferulic acid (FA) content of *M*. × *giganteus* down to about 59.30% of the original, depending on the pretreatment severity (Table 1). A similar decrease in HCA content due to acid pretreatment of *M*. × *giganteus* has been observed in an earlier study [24]. This reduction is greatly beneficial to the solubilization of arabinoxylans, which facilitates opening up of cell wall structure and enhancement of enzyme activity.

### Enzymatic digestibility of chemically pretreated *M*. × *giganteus*

**Figure 1:**
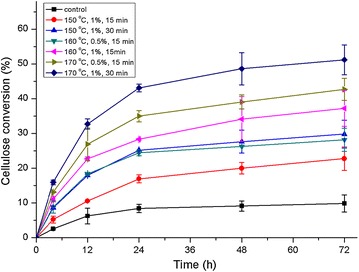
Enzymatic hydrolysis profile of dilute acid-pretreated *M.* × *giganteus*.* Error bars* indicate standard deviation.

Enzymatic saccharification assays were employed to probe differences in sugar release from *M.* × *giganteus* samples. Enzymatic digestibility correlates strongly with treatment effectiveness and is an excellent probe for the accessibility of cellulose to depolymerization catalysts [30]. Figure 1 compares the glucan release from samples before and after DAP under gradient conditions up to 72 h of enzymatic hydrolysis. The raw materials displayed fairly low saccharification kinetics reaching a peak value 11.80%, which greatly highlighted the need to overcome cell wall recalcitrance. The presence of intact lignin polymer and possible coating effect of hemicelluloses in the rigid cell wall severely impeded enzymatic digestion. In contrast, the treated samples displayed distinctly superior glucan conversion. Among the factors influencing cellulose digestibility of recovered solids, temperature was most significant, followed by acidic strength and residence time, as the analysis of chemical composition would suggest. *M.* × *giganteus* solids pretreated at 170°C were more acquiescent to enzymatic digestion than at 150°C. At 160°C, an increase in the strength of acidic solution from 0.5 to 1% resulted in 9% promotion in the release of total sugar. Pretreating samples with 1% H_2_SO_4_ for 30 min at 170°C, followed by enzymatic degradation, generated the highest sugar yield (51.20%). Although the influence of microscopic and conformational changes of biomass upon DAP have been discussed, changes in cell wall ultrastructure and spatial distribution of cell wall components in *M*. × *giganteus* and their relationship to cellulose digestibility have not yet been reported. In our study, multimodal imaging was performed on two representative samples pretreated at 160°C, 0.5% H_2_SO_4_ for 15 min and 170°C, and 1% H_2_SO_4_ for 30 min to address this and to further elucidate the origins of the differences in glucan conversions.

### Anatomical changes in *M.* × *giganteus* cell walls

**Figure 2:**
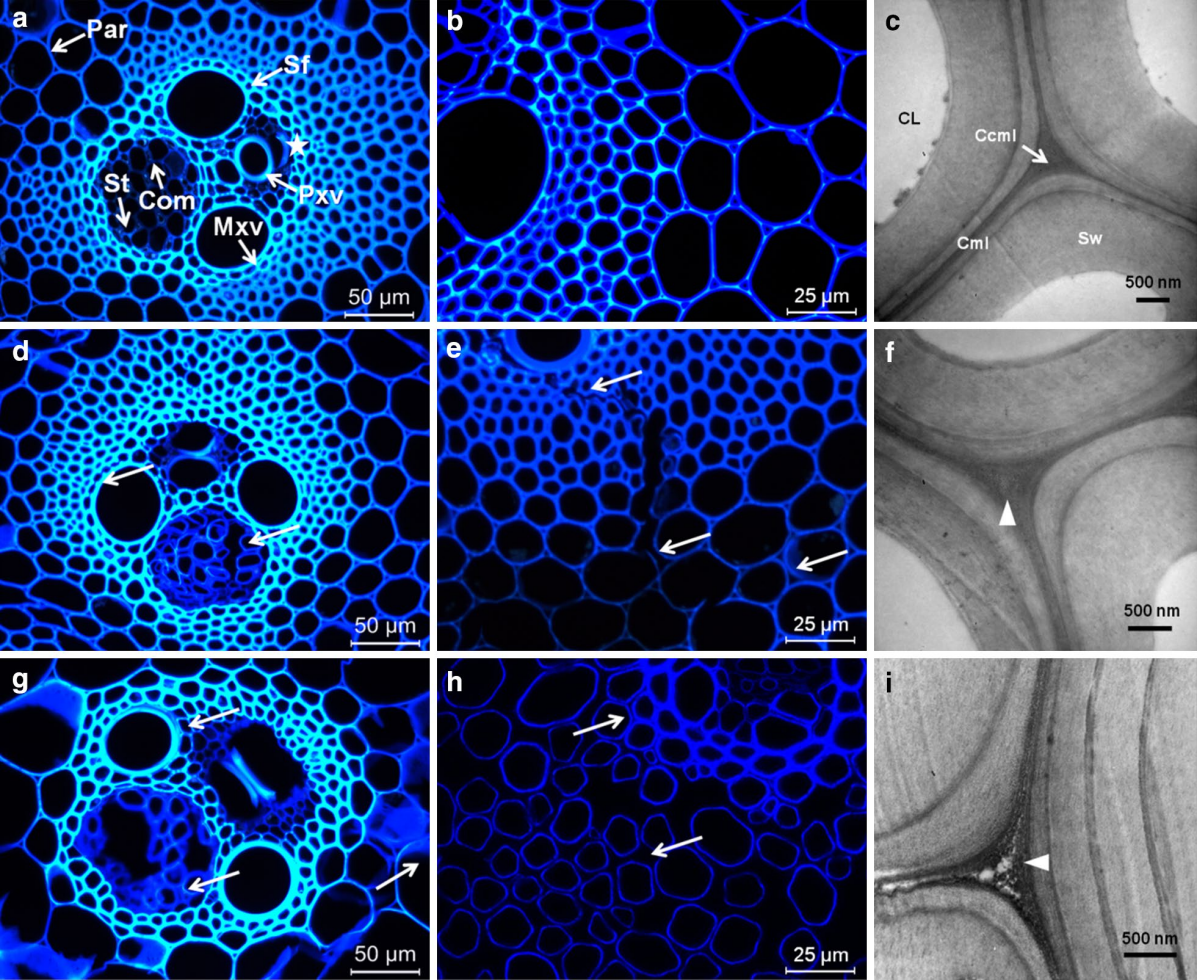
Multiscale imaging of *M.* × *giganteus* cell wall architecture by FM and TEM. FM images showed intact clusters of cells in raw *M.* × *giganteus* tissues (**a**, **b**) with trace evidence of mechanical damage near Pxv from the cutting process (**a**, *asterisk*). The ultrastructure of Sf including compound cell corner (Ccml), compound middle lamella (Cml), secondary wall (Sw) and cell lumen (CL) were observed by TEM (**c**). In samples pretreated at 160°C, 0.5% H_2_SO_4_ for 15 min, individual cell walls were shown to be separated, particularly at the St and Com regions (**d**, *white arrows*) and at the Sf–Par boundaries (**e**, *white arrows*). A TEM scan of Sf presented lighter staining in the Ccml indicating lower density in these regions (**f**, *white triangle*). Samples pretreated at 170°C, 1% H_2_SO_4_ for 30 min exhibited many crushes of broken cells (**g**). Increased disjoining of Par walls from the Cml can be clearly distinguished at higher magnifications (**h**, *white arrows*). Additionally, intercellular spaces at the Ccml of Sf were gradually generated, resulting from the removal of hemicelluloses and lignin (**i**, *white triangle*). *Sf* sclerenchyma fibers, *Par* parenchyma, *Pxv* protoxylem vessel, *Mxv* metaxylem vessel, *St* sieve tube, *Com* companion cell.

Figure 2 illustrates the morphology and layered structure of *M.* × *giganteus* cell walls as a function of acid treatment severity by multi-scale imaging strategies. FM was used to investigate the cell wall structure in vascular bundles. *M.* × *giganteus* culm tissues were well organized, consisting of several cell types, including sclerenchyma fiber (Sf) surrounding the vascular bundles, parenchyma (Par), protoxylem vessel (Pxv), metaxylem vessel (Mxv), sieve tube (St) and companion cell (Com). Prior to treatment, vascular bundle tissues appeared intact (Figure 2a, b) with trace evidence of mechanical damage near Pxv from the cutting process (asterisk in Figure 2a). After pretreatment under moderate condition (160°C, 0.5% H_2_SO_4_ for 15 min), the samples showed separation of cell walls in many locations (Figure 2d, e), particularly at St and Com regions (white arrows in Figure 2d) and at Sf–Par boundaries (white arrows in Figure 2e), which indicated loosening of the original structure. With increasing pretreatment severity, vascular bundles displayed more pronounced alterations to tissues, such as sucrose-storing Par; many cells were crushed and broken (Figure 2g). Cell separations among neighboring Par cells in the treated biomass were more clearly visible in higher magnification images (white arrows in Figure 2h), which may be attributable to effective depolymerization of hemicelluloses and removal of lignin and pectin from P and Cml regions. In alfalfa, the deposition and distribution of pectin conforms to the patterns of lignin in the Ccml, where much of the pectin in cell walls is located and lignification is initiated [31]. In addition, the pectic arabinogalactans are reported to be removed concurrently with lignin during delignification of lupin upon chemical treatments [32, 33]. A recent study by DeMartini et al. [34] employing a novel glycome profiling technique on *Populus* biomass during hydrothermal pretreatment demonstrates significant loss of pectic and arabinogalactan epitopes corresponding to the disintegration of lignin–polysaccharide linkages. Briefly, the disjoining of cell walls likely enhanced the exposure of cellulose microfibrils and availability of more active surface area, consequently increasing the cellulose digestibility of the treated biomass as shown earlier.

Thick-walled Sf cells were the main target tissues in *M*. × *giganteus* for polysaccharides for enzymatic hydrolysis. The effect of DAP on the ultrastructural features of Sf cells was investigated using TEM in combination with KMnO_4_ staining of cross sections of samples before and after pretreatment. This staining contrasts lignin, and various cell wall layers can be readily differentiated under TEM based on differences in lignin concentration [35]. Cml and sub-layers of Sw were clearly distinguished in the native *M*. × *giganteus* cell walls (Figure 2c). The layered structure of cell walls after mild treatment (160°C, 0.5% H_2_SO_4_ for 15 min) closely resembled that in the control samples (Figure 2f). However, the Ccml of Sf appeared perturbed displaying reduced density as evidenced by the light and sparsely stained regions (arrowheads in Figure 2f), in contrast to the relatively dark and homogeneously stained Ccml in the native cell walls (Figure 2c). Differences were more pronounced at elevated pretreatment condition (170°C, 1% H_2_SO_4_ for 30 min); intercellular spaces developed in Ccml regions (white triangle in Figure 2i) stemming from the removal of hemicelluloses and lignin, a finding not reported previously. TEM images further verified the existence of some dark globular and irregular particles in the original lignin-rich Ccml as well as the thin pit membrane (Pm) (see Additional file 1: Figure S2a–c). Aggregation of droplets in cell corners adjacent to Cml suggests coalescence of lignin, which appeared to migrate from the central Ccml into Cml regions. The presence of coalesced lignin in the Cml region common to Sf and Par indicates preferential diffusion of lignin into the Sw of Par compared to more lignified Sf (see Additional file 1: Figure S2d).

We propose that in addition to being removed, lignin migrates from Ccml into Cml regions, as well as from outer to inner cell wall regions, which can provide greater contact surface area for diffusion. It has been reported that when the pretreatment temperature exceeds the glass transition temperature for lignin (120–200°C), lignin-based components change their native aggregation forms and may be forced to coalesce into small droplets (10–100 nm) because of phase separation from an aqueous environment [36, 37]. Once in a more fluid state, the movement of lignin is plausible within the confines of the cell wall matrix. These features combined with the capillary effect contribute to the migration of lignin within cell walls. Selig et al. [38] reported that upon cooling after treatment, coalesced lignin could solidify and either become trapped within the wall layers or settle out of the bulk liquid, potentially depositing back onto the biomass surface. This behavior may be an alternative interpretation for the accumulation of droplets near the Ccml and Pm, further confirming previous results that dense spheres were aggregated in cell corners and pits and delamination zones revealed [36]. It should be noted here that the pretreated *M.* × *giganteus* samples were vigorously washed and dehydrated with ethanol prior to embedding, which may have removed spherical formations from the Sw surface. For a more definitive proposal for the mechanisms of lignin migration within cell walls, more rigorous experiments are required. Nevertheless, the movement and relocalization of lignin helps explain the presence of pores (increased cell wall porosity), which can facilitate enzyme penetration.

**Figure 3:**
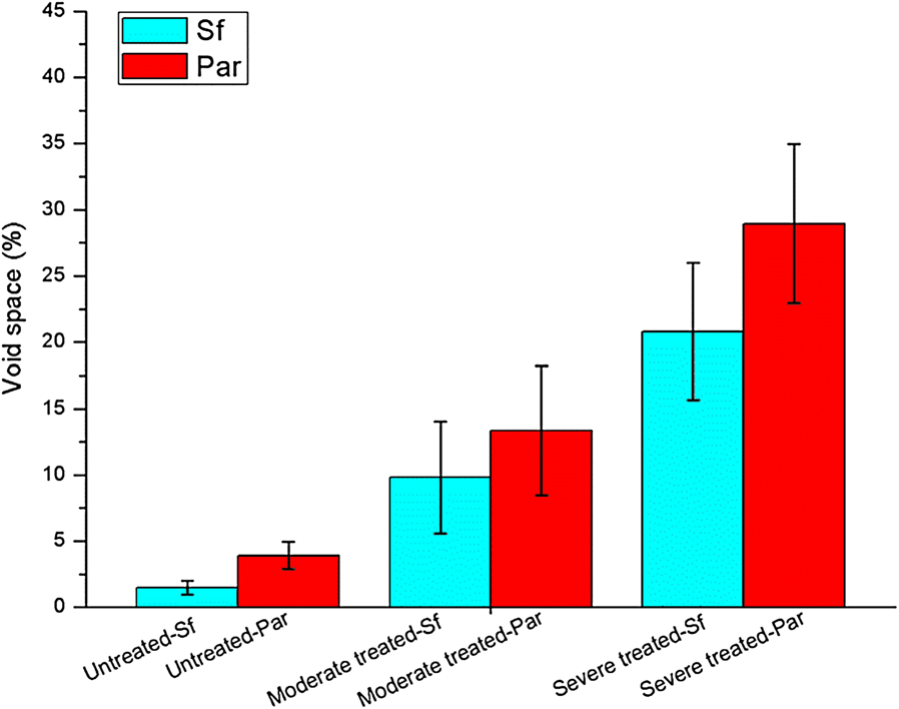
Intra-cell wall void space. Dilute acid pretreatment caused an increase in void spaces of Sf and Par revealing a loosening structure of *M.* × *giganteus* cell walls after pretreatment. Moderate treatment, 160°C, 0.5% H_2_SO_4_ for 15 min; severe treatment, 170°C, 1% H_2_SO_4_ for 30 min.

KMnO_4_ staining of ultrathin sections proved useful in visualizing also other anatomical changes in treated samples. Cml and boundaries of sub-layers of Sw appeared darker than other cell wall regions. Decreased staining of bulk of the cell wall reflects deconstruction and consequent loosening of cell wall structure. The extent of this loosening in Sf and Par tissues was quantified by calculating the intra-cell wall void spaces directly from TEM images (details of calculation included in Additional file 1: Figure S3). The results summarized in Figure 3 clearly suggest that DAP leads to the formation of more intra-cell wall void spaces in the treated samples subjected to harsher treatment (20.8 ± 5.2% for Sf and 29.0 ± 6.0% for Par) compared to native cell walls (1.5 ± 0.5% for Sf and 3.9 ± 1.1% for Par). It has also been suggested earlier that cell wall porosity in sugarcane bagasse increased due to oxalic acid pretreatment [39]. Though there may be discrepancies in data among studies undertaken due to variations in materials and cell wall characteristics, the described changes help explain the basis for enhanced enzymatic digestibility of treated *M.* × *giganteus*. DAP may not only open up the rigid cell wall structures, generating more surface area and creating more pore spaces, but also lower the nonproductive bindings of enzymes to lignin. In the context of enzymatic hydrolysis results, microstructural changes assist in explaining why DAP can promote release of sugars from cell walls.

### Immunogold labeling of xylans in *M.* × *giganteus* cell walls

The main effect of dilute acid pretreatment is to remove the coating hemicelluloses from cellulose microfibril surfaces, thus enhancing the accessibility of enzymes to cellulose. Xylan, the main hemicelluloses in *M*. × *giganteus*, is hydrolyzed to xylose or xylo-oligomers during acid-based pretreatments. Although the work by Alonso-Simon and coworkers [40] provided detailed analysis of hemicelluloses degradation upon hydrothermal pretreatment, the study only reported the bulk chemical changes of wood meal, but not the cell wall-level information. In our work, monoclonal antibody LM10 directed toward the unsubstituted or low-substituted xylans of *M*. × *giganteus* were incubated with colloidal gold-conjugated secondary antibodies. The location of gold particles that could be readily visualized under TEM marked the position of xylans within cell walls during pretreatment. The labeling intensity of each sample was calculated using Image J™ analysis which provided valuable information on the degree of xylan degradation by acid catalysis.

**Figure 4:**
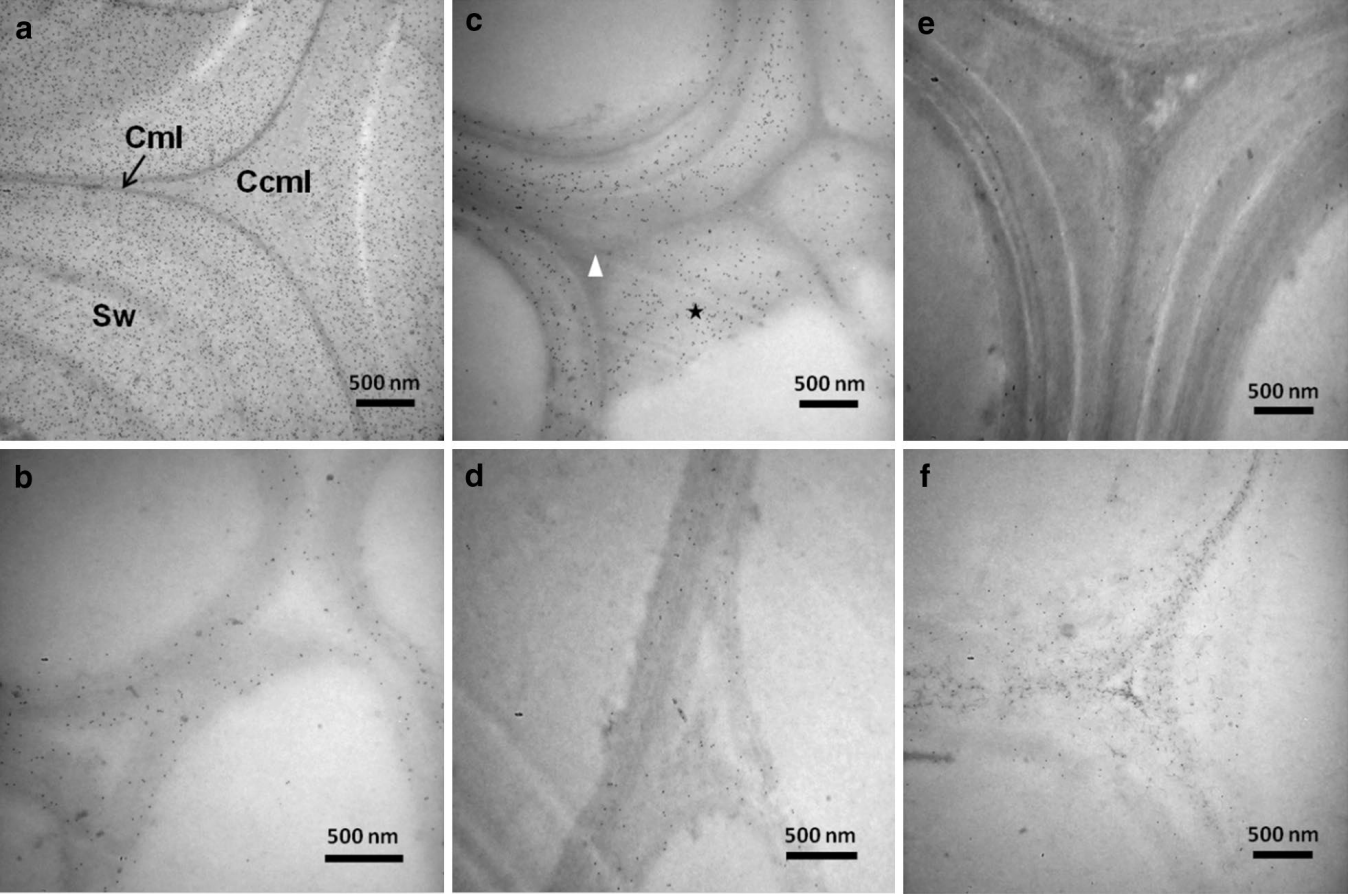
Immunogold localization of xylans by LM10 in Sf and Par of *M.* × *giganteus*. In raw materials, the majority of xylans were present in the Sw layer of Sf as a nearly continuous matrix rather than in the Ccml and Cml areas (**a**), while the Par walls showed a uniform distribution (**b**). In 160°C-treated samples, the xylans were clearly less abundant in the regions of Ccml and the adjacent outer Sw of Sf, but there was a retention near the middle Sw of corresponding cells (**c**, *asterisk*). The golden particles in the Par were much more sparse after acid treatment (**d**). In 170°C-treated samples, only infrequently were any xylans detected on the surface of Sf and Par (**e**, **f**). *Ccml* compound cell corner; *Cml* compound middle lamella; *Sw* secondary wall.

**Figure 5:**
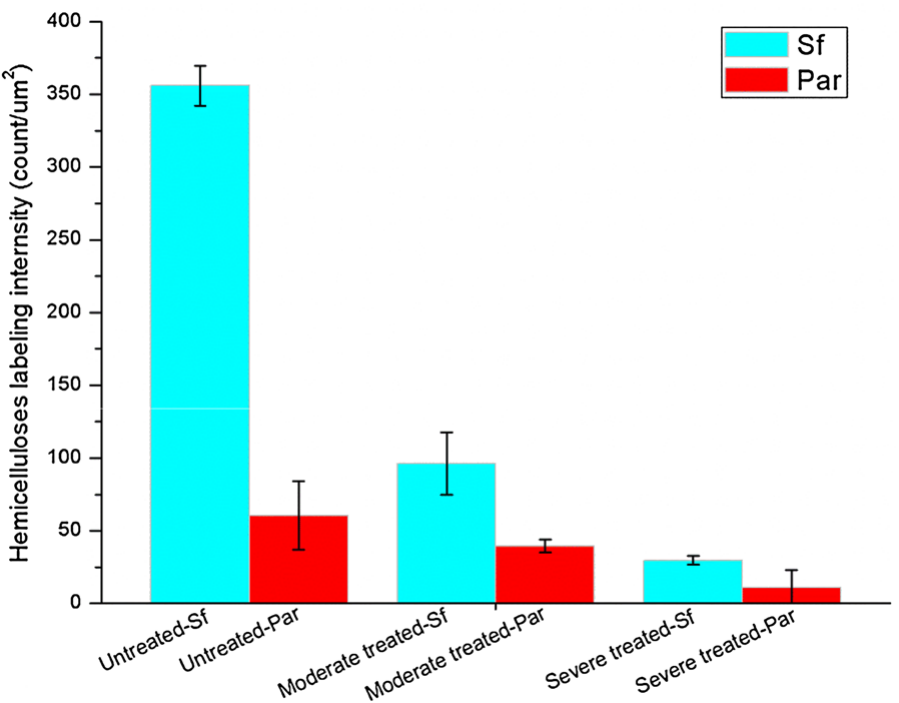
Hemicelluloses labeling density. Dilute acid pretreatment caused a reduction in the labeling intensity of hemicelluloses in Sf and Par of *M.* × *giganteus*. The severely treated samples at 170°C, 1% H_2_SO_4_ for 30 min displayed significantly more hemicelluloses degradation than the moderately treated samples at 160°C, 0.5% H_2_SO_4_ for 15 min.

In the native *M*. × *giganteus* samples, the majority xylans were present in the Sw layer of Sf as a nearly continuous matrix (Figure 4a). Par walls showed a uniform distribution of xylans with a relatively lower labeling intensity (Figure 4b). Judging from TEM images, xylans concentration decreased significantly during DAP; interestingly though, not all cell wall regions responded similarly to the pretreatment. For the samples pretreated at 160°C, 0.5% H_2_SO_4_ for 15 min, the xylans antibody signals displayed a conspicuous decrease in the Ccml and adjacent outer Sw regions of Sf, but a retention near the middle Sw of corresponding cells (Figure 4c). Brunecky et al. [41] using immunofluorescence labeling investigated the reorientation pattern of xylans upon DAP, drawing a different conclusion that there was a dramatic loss of xylans from the center of the cell wall and an increase or retention in the Cml. This discrepancy may be attributed to differences in the raw materials and/or different stages of maturation. Despite this discrepancy, both immunolabeling techniques emphasized the selective degradation and redistribution of xylans within cell walls during DAP. Overall, the average labeling intensity in Sf and Par in moderately treated samples declined from 356 ± 14 and 60 ± 24 per μm^2^ to 96 ± 21 and 40 ± 5 per μm^2^, respectively (Figure 5). The samples subjected to harsher acid treatment (170°C, 1% H_2_SO_4_ for 30 min) showed a further reduction in xylan labeling (Figure 4e, f). Only infrequently were any xylans detected on the surface of Sf with a labeling density of 11 ± 3 per μm^2^. Labeling intensity also diminished in Par to 30 ± 12 gold particles per μm^2^ as the treatment severity increased (Figure 5).

As mentioned earlier, several factors contribute to recalcitrance of lignocellulosic biomass toward chemical and enzymatic treatments, many of which relate to the presence of hemicelluloses [22]. Hemicelluloses that cross-linked to lignin function as an adhesive matrix among cellulose microfibrils, severely restricting pretreatment solutions and enzymes from entering into cell wall pore channels. Inhomogeneous distribution patterns of hemicelluloses also negatively impact on the diffusion process. Solubilization of xylans in Sf and Par tissues freed the surfaces of microfibrils as well as increased cell wall porosity. The behavior of xylan release is accompanied by deacetylation. It is favorable for providing more sites for enzyme attack and reducing recalcitrance through the linearization of hemicelluloses, thereby improving the saccharification efficiency of the pretreated biomass [42, 43].

### Raman imaging of phenolics and cellulose distribution in *M.* × *giganteus* cell walls

**Figure 6:**
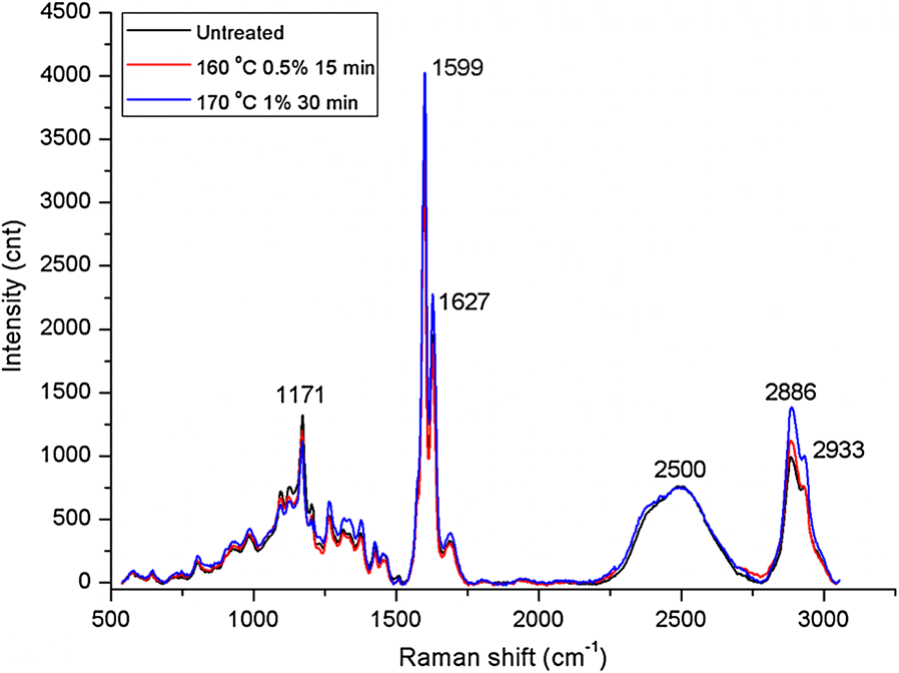
Average Raman spectra of raw and dilute acid-pretreated *M.* × *giganteus*. *Black line* untreated *M.* × *giganteus*; *red line* pretreated *M.* × *giganteus* at 160°C, 0.5% H_2_SO_4_ for 15 min; *blue line* pretreated *M.* × *giganteus* at 170°C, 1% H_2_SO_4_ for 30 min. The spectra were normalized by the peak intensity (height) of the O–D stretching band around 2,500 cm^−1^ for comparison.

Changes in the spatial distribution of phenolics and cellulose in the cell walls of *M*. × *giganteus* subjected to DAP were investigated in situ by CRM. Figure 6 shows the average Raman spectra collected from the Sw of Sf in the original and treated sections. The intensity curves at a range of Raman shifts were first normalized by the peak intensity (height) of the O–D stretching band around 2,500 cm^−1^ [44, 45]. Lignin and cellulose display prominent bands and thus can be easily detected in the regions of 1,575–1,620 cm^−1^, assigned to contribution of symmetric stretching of the aromatic ring, and 2,789–2,932 cm^−1^, known for the C–H and C–H_2_ stretching modes, respectively [46, 47]. According to our previous investigations, gramineous species possess a typical Raman band region between 1,152 and 1,197 cm^−1^ that was assigned to cinnamoyl ester in ferulate and *p*-coumarate groups, collectively referred to as HCA [48, 49].

**Figure 7:**
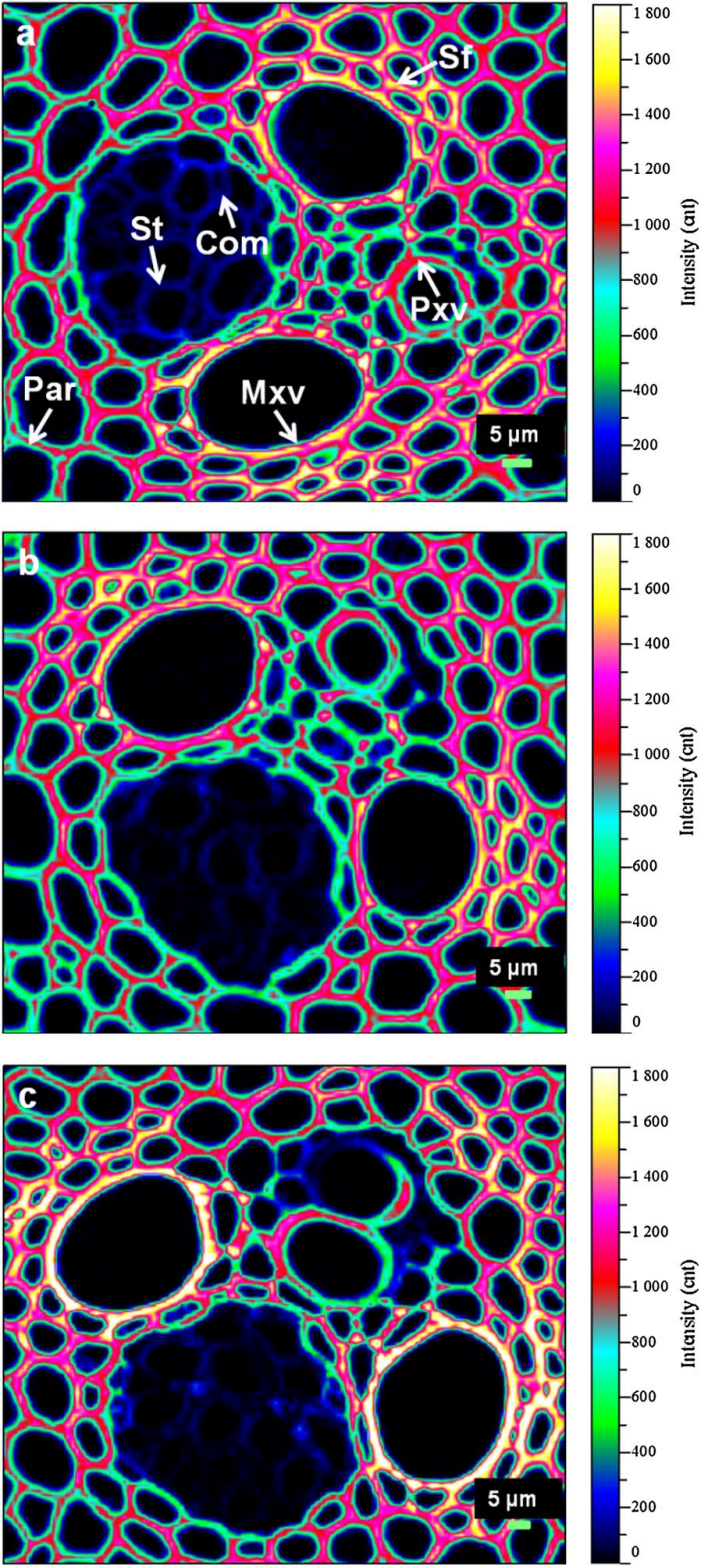
Raman images of lignin redistribution within *M.* × *giganteus* cell walls upon dilute acid pretreatment. **a** Untreated; **b** pretreated *M.* × *giganteus* at 160°C, 0.5% H_2_SO_4_ for 15 min; **c** pretreated *M.* × *giganteus* at 170°C, 1% H_2_SO_4_ for 30 min. *Sf* sclerenchyma fibers, *Par* parenchyma, *Pxv* protoxylem vessel, *Mxv* metaxylem vessel, *St* sieve tube, *Com* companion cell.

Raman images of lignin distribution were generated by integrating over the 1,575–1,620 cm^−1^ regions (Figure 7). The raw material had a heterogeneous distribution of lignin within various tissues, clearly displaying high intensity in Mxv, followed by Sf, and lowest in Pxv and Par (Figure 7a). Treatment of samples with aqueous acid caused redistribution of lignin, and the lignin content varied with pretreatment conditions. Based on the spectral analysis, the moderate pretreatment (160°C, 0.5% H_2_SO_4_ for 15 min) removed 5% lignin, while the harsher treatment (170°C, 1.0% H_2_SO_4_ for 30 min) resulted in greater lignin removal ca. 13%. Raman mapping technique helped visualization of lignin redistribution within specific tissues in selected portions of treated *M.* × *giganteus*. The lignin originally present in Pxv, Mxv and Sf walls largely resisted the moderate acid treatment, whereas the Par tissues were visibly delignified with evidence of a lower intensity at the bottom left of Raman images in Figure 7b. In comparison, severe pretreatment resulted in remarkable increase in the lignin signal intensity, especially in the Mxv (Figure 7c). One reason for this may be that with the removal of hemicelluloses during treatment, lignin was more exposed. Another possible explanation is that the dissolved lignin settled out from the bulk liquid and redeposited onto cell wall surfaces upon cooling after the pretreatment [38]. There are many factors that govern the extent of lignin relocalization following DAP, such as pretreatment severity, grass species and even the morphological regions tested. By scanning different areas, several vascular bundles were considerably conspicuous with an obvious reduction in lignin signal intensity at the same condition (see Additional file 1: Figure S4). Many investigations have suggested the effect of DAP on the fragmentation of lignin, usually leading to a slight delignification in biomass, the extent of which depends on the pretreatment severity [50–52]. Together, CRM imaging and TEM measurements provided more complete information on the removal, migration and relocalization of lignin resulting from DAP. Compared with the aforementioned compositional analysis, some differences in the residual lignin content may be attributed to the particle size of samples [53], from which thin sections were collected for Raman microspectroscopy. The intensity of lignin signal in Raman images was not directly related to the content of Klason lignin in samples. The primary focus of this section was on the dynamic relative distribution of lignin upon DAP, but not absolute lignin content. We suggest that the relocalization of lignin during DAP is as important as lignin removal in the context of glucan conversion, since both dramatically open up cell wall structures, leading to improved accessibility of cellulose to enzymes. Hydrothermal pretreatment has been reported to alter the role of lignin in biomass in terms of its association with pectins, arabinogalactans and xylans [34]. The work presented here supports that lignin content per se does not affect cellulose digestibility. Rather, the spatial distribution of lignin and its integration with other cell wall components appear to play a larger role.

**Figure 8:**
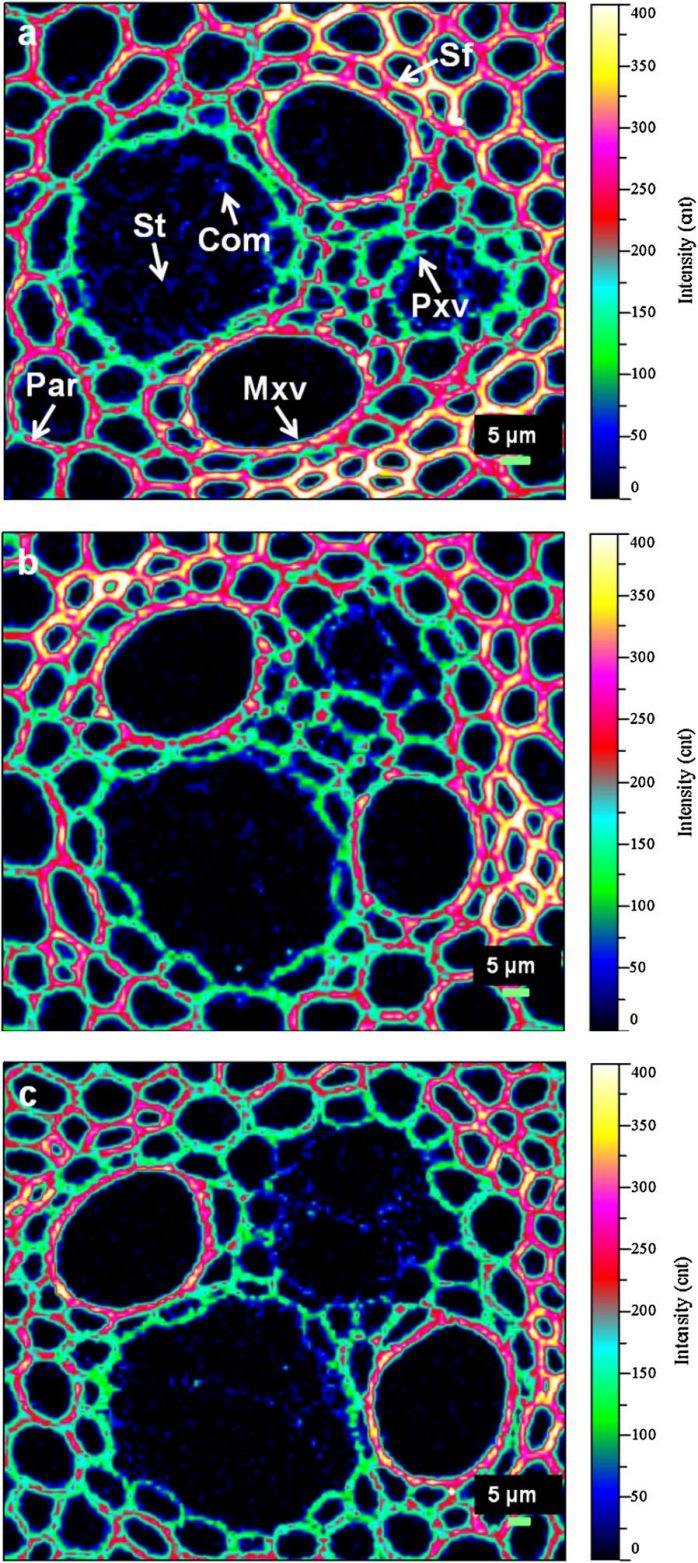
Raman images of hydroxycinnamic acids distribution within *M.* × *giganteus* cell walls upon dilute acid pretreatment. **a** Untreated; **b** pretreated *M.* × *giganteus* at 160°C, 0.5% H_2_SO_4_ for 15 min; **c** pretreated *M.* × *giganteus* at 170°C, 1% H_2_SO_4_ for 30 min. *Sf* sclerenchyma fibers, *Par* parenchyma, *Pxv* protoxylem vessel, *Mxv* metaxylem vessel, *St* sieve tube, *Com* companion cell.

In grass tissues, HCA plays an important role in cross-linking polymers into a cohesive network that contributes to biomass recalcitrance. By integrating over the band regions from 1,152 to 1,197 cm^−1^, HCA was visualized to be mainly accumulated within the Sf, Par and Mxv of *M.* × *giganteus* (Figure 8a). After DAP, obvious signal reduction in HCA was detected under both conditions employed, indicating acid-catalyzed degradation of this constituent (Figure 8b, c). The observation was much more pronounced in the case of treated samples at 170°C, 1% H_2_SO_4_ for 30 min. It has been reported that decreased ester-linked PCA/FA ratio is associated with increased forage digestibility in barley [54] and increased cellulose digestibility in switchgrass [55]. FA and PCA serve as bridges between lignin and hemicelluloses via ether and ester bonds forming lignin/phenolic–carbohydrate complexes [29]. Acid catalysis cleaves the ester linkages between FA and arabinose moiety of xylan chains and also efficiently disrupts the coumaric acid ester. A concomitant disruption of ether linkages between FA and lignin was simultaneously observed in the process. These modifications in pretreated *M.* × *giganteus* point to a looser association between lignin and arabinoxylans. It further facilitates hemicelluloses’ dissolution and phenolics’ redistribution, and thus increases surface areas and void spaces in pretreated samples.

**Figure 9:**
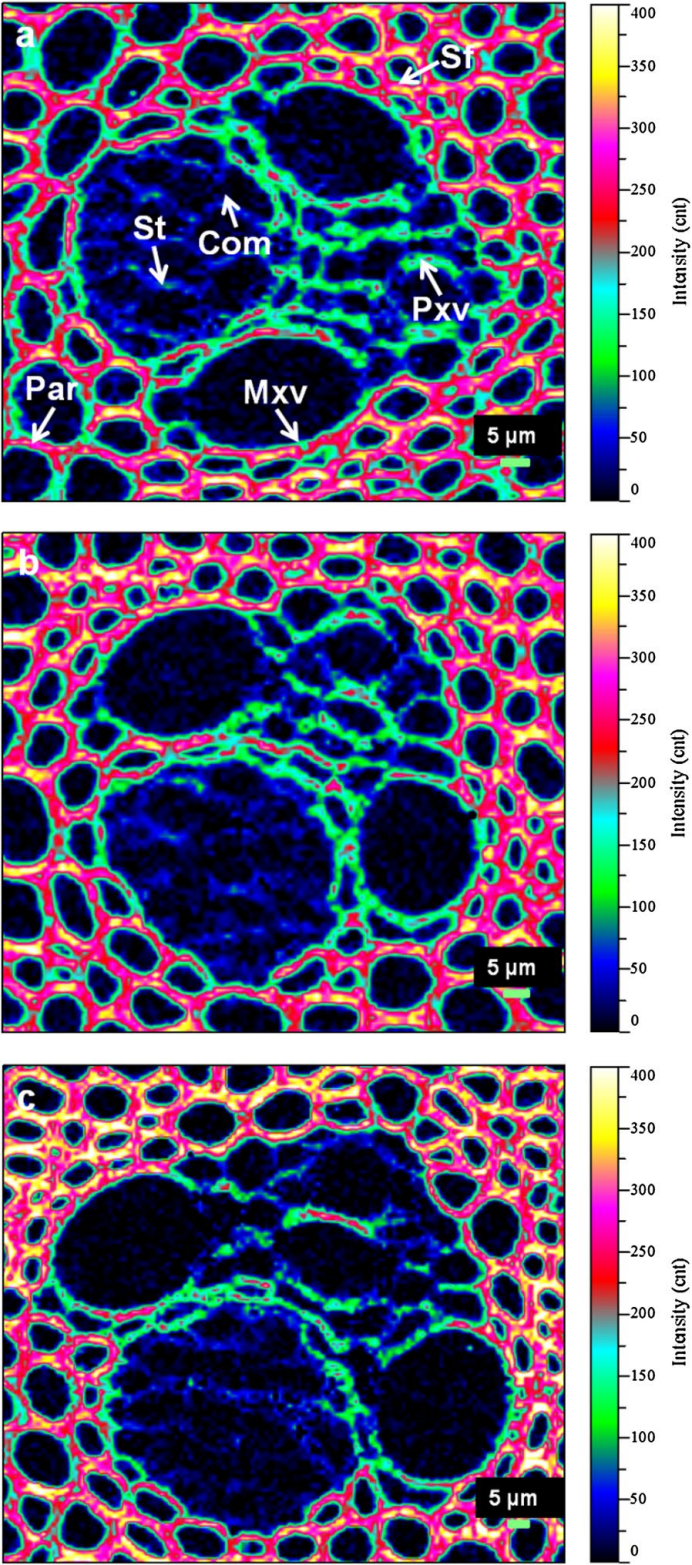
Raman images of cellulose distribution within *M.* × *giganteus* cell walls upon dilute acid pretreatment. **a** Untreated; **b** pretreated *M.* × *giganteus* at 160°C, 0.5% H_2_SO_4_ for 15 min; **c** pretreated *M.* × *giganteus* at 170°C, 1% H_2_SO_4_ for 30 min. *Sf* sclerenchyma fibers; *Par* parenchyma, *Pxv* protoxylem vessel, *Mxv* metaxylem vessel, *St* sieve tube, *Com* companion cell.

Cellulose distribution in the *M.* × *giganteus* was highlighted by integrating over the spectral range from 2,789 to 2,932 cm^−1^. The Sf of native materials with high brightness suggested accumulation of celluloses in these regions, contrary to Pxv and Mxv (Figure 9a). Though the 2,886 cm^−1^ band is admittedly favored from hemicelluloses, evaluation of Raman spectra of treated samples yet revealed an increase in this peak with diminution of hemicelluloses (Figure 4). The Raman imaging data also confirmed the results. Compared to Figure 9b, the cellulose concentration in samples treated with anabatic severity increased significantly, typically in the Sf (Figure 9c), which implies greater exposure of cellulose. Phenolics and hemicelluloses are known to coat cellulose microfibrils hindering chemical deconstruction of lignocellulosic cell walls [56, 57]. Xylans irreversibly absorbed on cellulose surface further reduce the action of cellulases [58]. Therefore, with the removal of a large portion of phenolics and hemicelluloses, cellulose cores were more exposed to be detected. The exposure of cellulose greatly facilitates enzymatic hydrolysis of cellulosic fractions in the treated biomass. This further explained the higher yield of fermentable sugars as illustrated in Figure 2. Holopainen-Mantila et al. [14] also suggested a similar effect on wheat straw cell walls after hydrothermal pretreatment.

## Conclusions

A combination of microscopic measurements and wet chemical analysis gives deeper insights into cell wall deconstruction in *M*. × *giganteus* subjected to DAP than was previously possible. Results indicated that dilute sulfuric acid pretreatment is an effective approach for improving enzymatic digestibility of plant biomass by altering the tissue’s anatomical and topochemical features in a temperature-dependent manner. The significantly modified Sf and Par tissues by DAP produced a heterogeneous substrate of semi-separated cells. Ccml and Sw regions of Sf firstly stepped into the breach, suggesting the rigid structure to be the main barriers for its effective deconstruction. In terms of topochemical changes, DAP induced selective solubilization of hemicelluloses and lignin migration within tissues that together facilitated loosening of cell wall structure. This treatment not only opened up the cell wall structure, rendering cell wall more porous, but also had an impact on the cleavage of lignin–carbohydrate linkages resulting from HCA removal. These alterations further enhanced the accessibility of enzymes to cellulose, as cellulose surfaces became more highly exposed. Although it is difficult to identify the most significant factors that influence the enzymatic digestibility due to the multitudinous changes that occurred simultaneously during pretreatment, the knowledge gained here hints at some possibilities that contribute to recalcitrance. This not only affords a platform from which more targeted studies can be undertaken to further evaluate the effect of specific characteristics, but can also assist researchers in bioengineering for efficient cell wall deconstruction using acid-based treatments with the aim of optimizing bioenergy yield.

## Methods

### Plant materials preparation

*M.* × *giganteus* was manually collected in July 2014 from a local experimental forest in Beijing Academy of Agriculture and Forestry Sciences, China. The leaves were removed, and the dried stems were ground and Soxhlet-extracted with benzene/alcohol (2:1, v/v) for 9 h. The extractive-free sample was oven dried at 40°C for 24 h, yielding a material with less than 2% moisture. For microscopic measurements, an *M.* × *giganteus* culm that was 2.3 m in length with a diameter at breast height of 7 mm was chosen. Approximately, 5 μm-thick cross sections were cut from the eighth internode of the culm using a sliding microtome. These thin sections were then used for dilute acid treatment. The chemicals used in this experiment were all purchased from Sigma-Aldrich (Shanghai, China).

### Dilute acid pretreatment

The sulfuric acid pretreatment was conducted in a batch mode using mineralization bombs equipped with Teflon cups (Parr). About 20 g extracted *M.* × *giganteus* samples were presoaked in 0.5 and 1.0% sulfuric acid solution at a ratio of 1:10 at room temperature for 2 h and then incubated at 150, 160 and 170°C for 15 and 30 min in an oil bath, respectively. After pretreatment, the recovered solid fractions were brought to neutral PH and stored at −20°C for enzymatic hydrolysis. The pretreated sections were washed thoroughly with de-ionized water to remove any acid before microscopic measurements.

### Chemical component analysis

The chemical component analysis of untreated and treated samples was performed according to NREL procedures LAP-002 [59]. The monomer sugars were analyzed by high-performance anion exchange chromatography (HPAEC) system (Dionex ICS 3000, USA) with pulsed amperometric detector, AS50 autosampler, the Carbopac™ PA-20 column (4 × 250 mm, Dionex) and the guard PA-20 column (3 × 30 mm, Dionex). Neutral sugars and uronic acids were separated in a 5 mM NaOH (isocratic; carbonate free and purged with nitrogen) for 20 min, followed by a 0–75 mM NaAc gradient in 5 mM NaOH for 15 min. Then the columns were washed with 2 mM NaOH to remove carbonate for 10 min, followed by a 5 min of elution with 5 mM NaOH to re-equilibrate the column before the next injection. The total analysis time was 50 min and the flow rate was 0.4 ml/min. The content of HCA was determined using the procedure outlined in Ref. [14].

### Enzymatic hydrolysis of *M.* × *giganteus*

The native and pretreated samples were enzymatically hydrolyzed in a 0.05 M sodium acetate buffer with a pH of 4.8 at a biomass loading of 10% (w/v) in an air-shaking incubator maintained at 50°C at 150 rpm for 72 h. Commercial cellulase (Novozyme) was employed at an activity of 20 FPU/g substrate for all samples. The reactions were monitored by taking 100 μL supernatant at specific time intervals, followed by deactivation of the enzymes in boiling water for 10 min and centrifugation at 10,000*g* for 5 min. The released monosaccharides were analyzed by HPAEC under the same conditions as those described above. The results are expressed as percentage of the total cellulose in the substrate. All assays were performed in duplicate.

### Fluorescence microscopy measurements

The original and treated transverse sections of *M.* × *giganteus* were dehydrated through a graded series of ethanol solution. Subsequently, the sections were mounted in glycerol and covered with a coverslip (0.17 mm thickness). Sections were examined with a Leica DM 2000 fluorescence microscope using an ultrapressure mercury lamp for illumination. The excitation wavelength was 435–480 nm and the emission wavelength at 495–600 nm was used for imaging lignin autofluorescence.

### Transmission electron microscopy measurements

Samples were fixed in 4% paraformaldehyde buffered in 0.1 M sodium cacodylate buffer under vacuum. After dehydration with graded ethanol series (30, 50, 70, 90%, twice for 100% ethanol), samples were infiltrated with increasing concentrations of LR White resin (30, 50, 70, 90%, thrice for 100% resin, diluted in ethanol) under vacuum and then transferred to gelatin capsules for resin polymerization by heating to 60°C overnight. Subsequently, transverse ultrathin sections were prepared from the embedded blocks using an ultramicrotome (Leica EMUC7) equipped with a diamond knife and mounted on copper or nickel grids. The grids were stained with 1% w/v KMnO_4_ (prepared in 0.1% sodium citrate) for 3 min to selectively stain for lignin at room temperature. Images were taken with a transmission electron microscope (TEM, JEM1220, Japan) at an accelerating voltage of 80 kV.

### Confocal Raman microscopy measurements

The native and pretreated sections were placed on a glass slide with a drop of D_2_O which was set as the internal standard and then covered with a coverslip (0.17 mm thickness) for Raman detection. Raman spectra were acquired at room temperature using a LabRam Xplora exquisite full-automatic confocal Raman microscope (Horiba Jobin–Yvon), equipped with an MPlan 60× oil immersion microscope objective (Olympus, NA = 1.35) and a 532-nm laser in the visible wavelength range. The wavenumber ranged from 3,200 to 600 cm^−1^ with a confocal aperture at 200 μm and slit width at 100 μm. The lateral resolution of our system was approximately 0.5 μm, which is lower than the theoretical prediction (0.61λ/NA ≈ 240 nm). For imaging, an integration time of 2 s was chosen and every pixel corresponded to one scan acquired every 0.5 μm by averaging 2 s cycles. The Labspect 5 software was used for spectra and image processing and analysis. The Raman images showing the distribution of a certain constituent were calculated by integrating the corresponding Raman band region.

### Immunogold labeling

After suspension of the grids in buffer A (pH 8.2 Tris-buffered saline containing 1% bovine serum albumin) for 30 min at room temperature, the grids were incubated with LM10 (1:20 dilution in buffer A) for 2 days at 4°C. After washing with buffer A, the grids were incubated with goat anti-rat secondary antibody labeled with 10-nm colloidal gold particles (BB International, UK) for 4 h at 35°C for the LM10 antibody (1:20 dilution in buffer A). For the control, some sections were also incubated only with secondary antibody. Finally, the grids were washed by buffer A, followed by distilled water. The grids were post-stained with 2% uranyl acetate and examined under a transmission electron microscope (TEM, JEM1220, Japan).

### Image analysis

Hemicellulose density within cell walls was measured by counting gold particles in grayscale thresholded images using Image J™ (NIH, Bethesda, MD). Over 50 μm^2^ of cell walls were analyzed across ten micrographs for each treated sample.

## Additional file

Additional file 1: Supplementary supporting data. **Figure S1**. Mass loss of dilute acid pretreated *M. *× *giganteus* under various conditions. **Figure S2**. TEM micrographs of *M. *× *giganteus* Sf after pretreatment with 1% H_2_SO_4_ at 170°C for 30 min. **Figure S3**. Explanation of void space calculation. **Figure S4**. Raman image of lignin distribution in treated *M. *× *giganteus* at 170°C, 1% H_2_SO_4_ for 30 min calculated by integrating over the spectral range from 1575 to 1620 cm^−1^.

## Abbreviations

DAP: dilute acid pretreatment
FM: fluorescence microscopy
TEM: transmission electron microscopy
CRM: confocal Raman microscopy
P: primary wall
Sw: secondary wall
Ccml: cell corner middle lamella
Cml: compound middle lamella
DM: dry matter
H_3_O^+^: hydronium ions
HCA: hydroxycinnamic acids
PCA: *p*-coumaric acid
FA: ferulic acid
Sf: sclerenchyma fiber
Par: parenchyma
Pxv: protoxylem vessel
Mxv: Metaxylem vessel
St: sieve tube
Com: companion cell
