# Supplementary material for: Visualization of Miscanthus × giganteus cell wall deconstruction subjected to dilute acid pretreatment for enhanced enzymatic digestibility
Source: Biotechnol Biofuels. 2015 Jul 25;8:103. doi: 10.1186/s13068-015-0282-3 (PMC4513789; doi:10.1186/s13068-015-0282-3)
Supplement: Additional file 1: — Supplementary supporting data. Figure S1. Mass loss of dilute acid pretreated M. × giganteus under various conditions. Figure S2. TEM micrographs of M. × giganteus Sf after pretreatment with 1% H2SO4 at 170°C for 30 min. Figure S3. Explanation of void space calculation. Figure S4. Raman image of lignin distribution in treated M. × giganteus at 170°C, 1% H2SO4 for 30 min calculated by integrating over the spectral range from 1575 to 1620 cm−1. [file 13068_2015_282_MOESM1_ESM.pdf]

**Additional file for**

# Visualization of *Miscanthus* x *giganteus* cell wall deconstruction subjected to dilute acid pretreatment for enhanced enzymatic digestibility

Zhe Ji [1, 2], Xun Zhang [1, 2], Zhe Ling [1, 2], Xia Zhou [1, 2], Shri Ramaswamy [3] and Feng Xu [1, 2*]

[1] Beijing Key Laboratory of Lignocellulosic Chemistry, Beijing Forestry University, Beijing, 100083, China

[2] Ministry of Education Key Laboratory of Wooden Material Science and Application, Beijing Forestry University, 100083, Tsinghua East Road, Beijing, China

[3] Department of Bioproducts and Biosystems Engineering, Kaufert Laboratory, University of Minnesota, Saint Paul, MN 55108, USA

*Corresponding author. Tel/fax: +86 10 62337993; E-mail address: xfx315@bjfu.edu.cn

## Calculation of Intra-Cell Wall Void Space

Increases in cell wall porosity due to dilute acid pretreatment were quantified by processing TEM micrographs to threshold intra-wall void spaces into regions from which the size and shape of voids could be measured. Six regions of interest (ROI) from each of five different images of the three samples were analyzed to determine void space. To select a threshold that would distinguish void spaces from intact cell wall regions, the mean and standard deviation of pixel intensity values within a ROI containing only void space (Figure S3a *), such as the cell lumen, were measured. The threshold was then determined as the pixel intensity value which was two standard deviations above the mean pixel value of the designated void region. Calculations were based on the following formula:

$$T = \bar{a}+2\sigma_v$$

Where $T$ is the threshold value, and $\bar{a}$ and $\sigma_v$ are respectively mean and standard deviation of pixel intensity values from a known void region. Examples of measured ROIs and the binary images determined using this thresholding method are given in Figure S3.

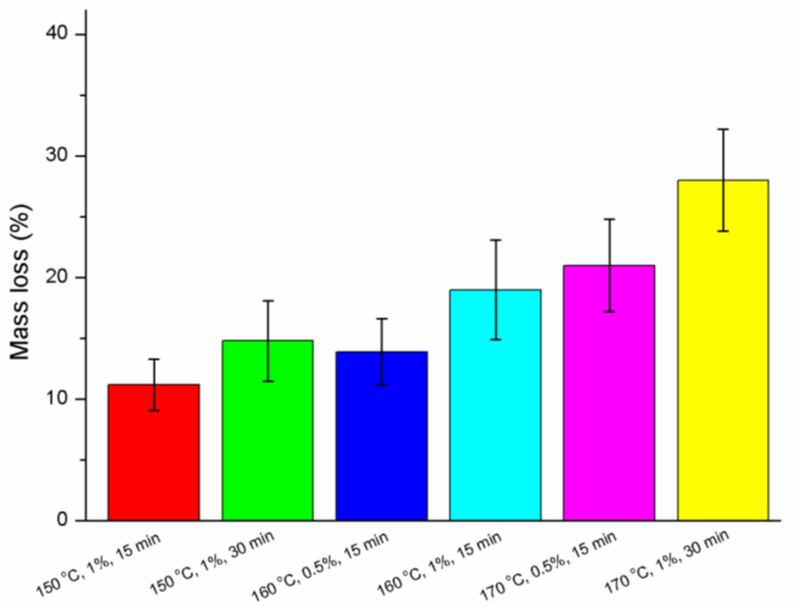


**Figure S1 Mass loss of dilute acid pretreated *M.* x *giganteus* under various conditions.**

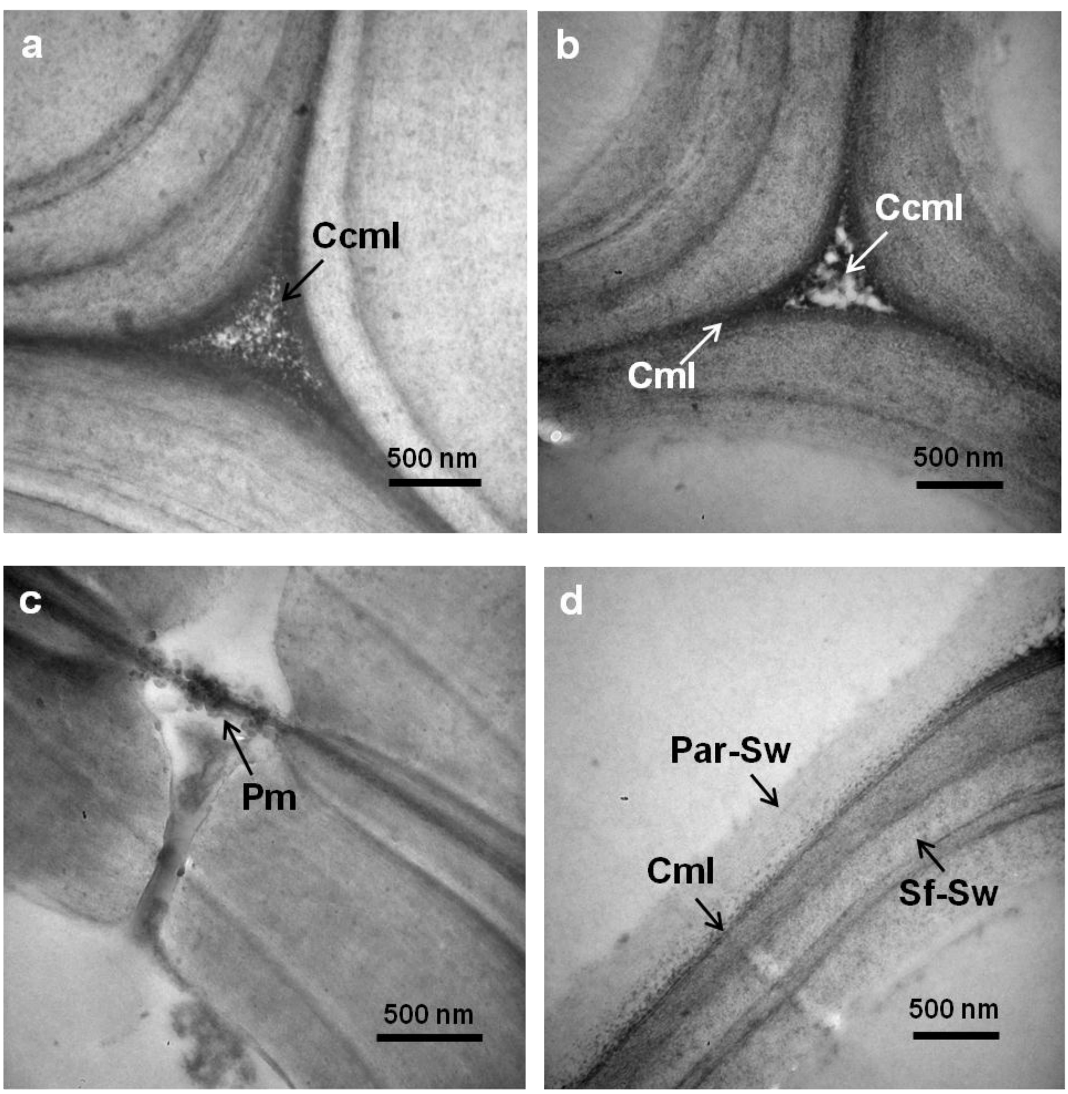


**Figure S2 TEM micrographs of *M.* x *giganteus* Sf after pretreatment with 1% $H_2SO_4$ at 170 $^oC$ for 30 min. (a-b)** The Ccml regions were visibly absent and aggregation of droplets were seen to migrate from the Ccml to the adjacent Cml; **(c)** some dark globular and irregular formations were accumulated in the thin Pm. **(d)** at the shared Cml the droplets preferentially diffused into the Sw of Par compared to the more lignified Sf. Ccml, compound cell corner; Cml, compound middle lamella; Sw, secondary wall; Pm, pit membrane.

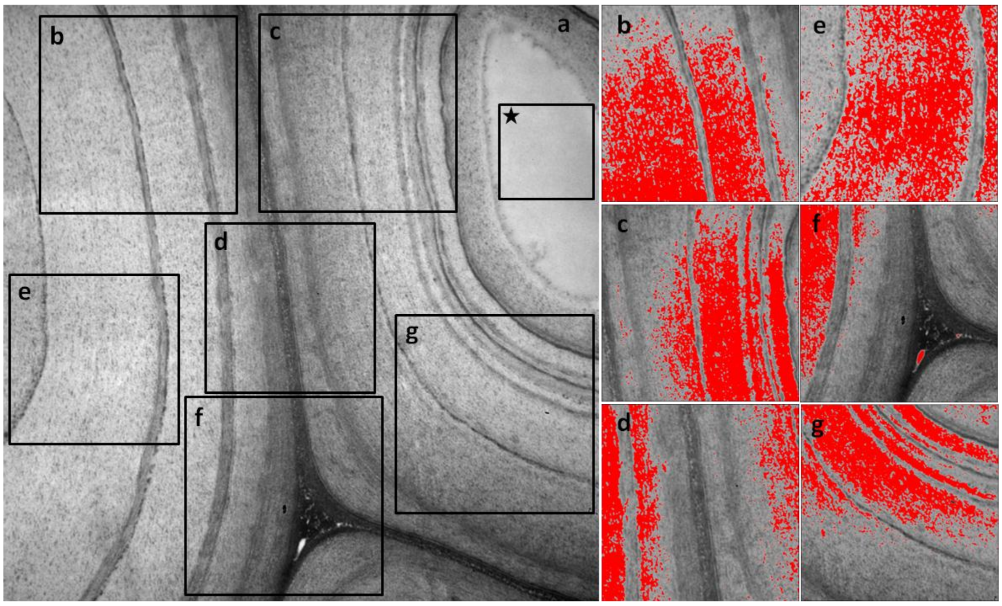


**Figure S3 Explanation of void space calculation. (a)** Original TEM image of dilute acid treated Sf of *M.* x *giganteus*. The ROI denoted with asterisk designates the known void region from which the threshold value was calculated; **(b-g)** examples of intra-cell wall ROIs selected from the original image. The right panels show the effect of applying the calculated threshold to the ROIs and the resulting void space values (shown in red).

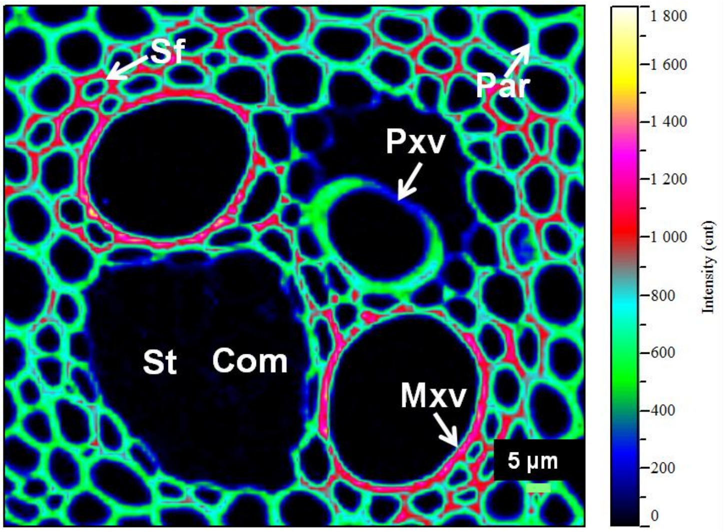


**Figure S4 Raman image of lignin distribution in treated *M.* x *giganteus* at 170 $^{o}$C, 1% $H_2SO_4$ for 30 min calculated by integrating over the spectral range from 1575 to 1620 cm$^{-1}$.** It showed the distribution of lignin with decreased brightness compared to the native sample, indicating lignin removal upon dilute acid pretreatment. Sf, sclerenchyma fibers; Par, parenchyma; Pxv, protoxylem vessel; Mxv, metaxylem vessel; St, sieve tube; Com, companion cell.
